# The N-terminal tail of the hydrophobin SC16 is not required for rodlet formation

**DOI:** 10.1038/s41598-021-04223-6

**Published:** 2022-01-10

**Authors:** Kathleen L. Vergunst, David N. Langelaan

**Affiliations:** Department of Biochemistry & Molecular Biology, Dalhousie University, Halifax, NS Canada

**Keywords:** Protein folding, Atomic force microscopy, NMR spectroscopy, X-ray crystallography, Biomaterials - proteins

## Abstract

Hydrophobins are small proteins that are secreted by fungi, accumulate at interfaces, modify surface hydrophobicity, and self-assemble into large amyloid-like structures. These unusual properties make hydrophobins an attractive target for commercial applications as green emulsifiers and surface modifying agents. Hydrophobins have diverse sequences and tertiary structures, and depending on the hydrophobin, different regions of their structure have been proposed to be required for self-assembly. To provide insight into the assembly process, we determined the first crystal structure of a class I hydrophobin, SC16. Based on the crystal structure, we identified a putative intermolecular contact that may be important for rodlet assembly and was formed in part by the N-terminal tail of SC16. Surprisingly, removal of the N-terminal tail did not influence the self-assembly kinetics of SC16 or the morphology of its rodlets. These results suggest that other regions of this hydrophobin class are required for rodlet formation and indicate that the N-terminal tail of SC16 is amenable to modification so that functionalized hydrophobin assemblies can be created.

## Introduction

Hydrophobins are a group of small, globular proteins produced and secreted by filamentous fungi that self-assemble into larger, amphipathic structures at hydrophilic-hydrophobic interfaces^1–7^. These assemblies create durable, protective, and water repellent coatings that fulfill various biological roles for fungi such as decreasing the surface tension of water, allowing hyphae to grow through the water’s surface^8,9^, and acting as water repellent coatings on fungal fruiting bodies and spores^10^. Hydrophobins also function as adhesion points for fungi by assembling between the cell wall and hydrophobic surfaces, which plays an important role in the pathogenicity of rice blast fungus (*Magnaporthe grisea*)^11,12^.

Due to their unusual properties, there is considerable interest in using hydrophobins as emulsifiers, surface modifiers, and protective coatings^2,13–17^. This includes applications such as increasing the bioavailability of water-insoluble drugs, modifying surfaces of medical implants, and as foam stabilizers to improve shelf life and food texture^7^. In order to develop hydrophobins for particular applications, studies are required to determine what influences the properties of hydrophobin assemblies and which regions of hydrophobins are necessary for function.

The amino acid sequence and molecular structure of hydrophobins are diverse but there are some characteristic features, including eight cysteine residues that form four conserved disulphide bonds that are separated by three loops (L_1_–L_3_, also referred to as the C3–C4, C4–C5, and C7–C8 intercysteine loops)^18^. Based on the spacing between cysteine residues, the pattern of hydrophobic patches, and the properties of their assemblies^6,19^ hydrophobins are broadly divided into two classes (class I and class II). Class I hydrophobins assemble into extremely stable amyloid-like structures called rodlets that resist denaturation by detergent, heat, and acid^2^. They are produced by Ascomycota and Basidiomycota fungi, with constituent hydrophobins having little sequence or inter-cysteine sequence length conservation^6^. Conversely, class II hydrophobins assemble into less stable films, do not form rodlets, are only secreted by Ascomycota fungi, and share a more conserved sequence. Based on phylogenetic analysis of hydrophobin sequences, class I sequences can be further separated into those originating from the Ascomycota (class IA) and Basidiomycota (class IB) fungi^6,20^.

Class IB hydrophobins have a greater degree of sequence conservation than class IA hydrophobins, suggesting that they may follow a common assembly mechanism. Current models of rodlet assembly have been developed by studying the class IA hydrophobin EAS (from *Neurospora crassa*) and involve L_3_ undergoing a structural transition from a disordered loop to a β-strand, which then oligomerizes with L_3_ of other EAS molecules into amyloid-like rodlets^21^. However, this model is not compatible with the nuclear magnetic resonance (NMR)-derived structure of the class IB hydrophobin SC16 (from *Schizophyllum commune*), since in SC16 L_3_ only consists of four residues which are structured as a β-turn. This makes it unlikely that L_3_ of SC16 can undergo large conformational changes during rodlet assembly, meaning that rodlet assembly likely involves other regions of SC16.

To investigate possible assembly mechanisms of SC16 we used X-ray crystallography to determine its atomic-resolution crystal structure, with the rationale that crystal contacts may provide insight into how SC16 monomers may associate with each other. We noted a large crystal contact was formed that involved the N-terminal tail of SC16. We then used NMR spectroscopy to investigate the dynamics of this tail, and functional assays to investigate how this region of SC16 influences rodlet assembly.

## Results and discussion

### Crystallization of SC16 identifies a possible biological contact

**Figure 1:**
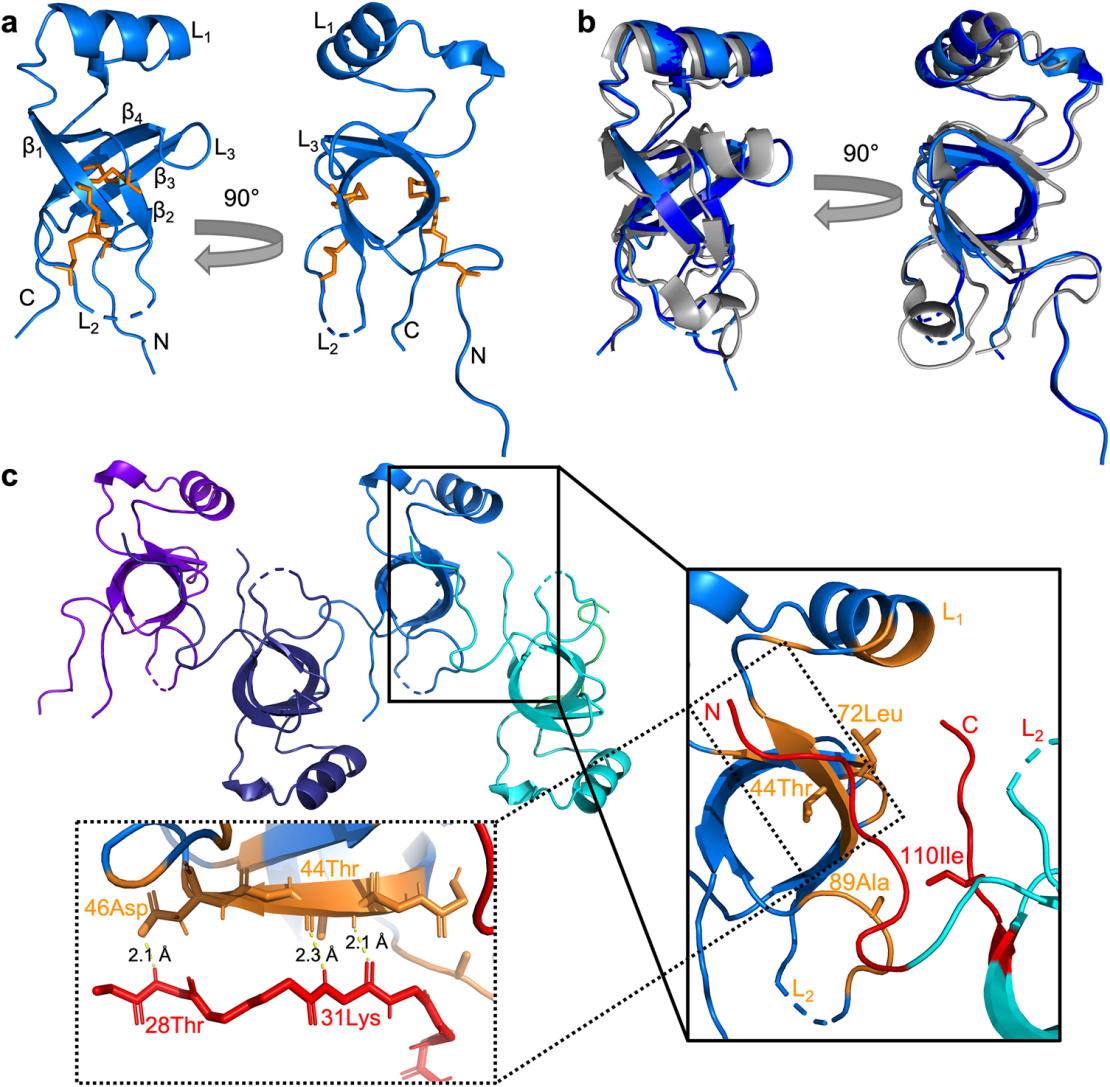
X-ray crystal structure of SC16. (**a**) A ribbon representation of SC16 crystallized in the C222_1_ space group (PDB ID: 7S86). Two different orientations are shown and the beta strands (β_1_ − β_4_), and loops (L_1_ − L_3_) are indicated. Conserved disulphide bonds are highlighted as orange sticks. (**b**) A superposition of SC16 crystalized in the C222_1_ (light blue) and P2_1_2_1_2 (PDB ID: 7S7S; dark blue) space groups with the NMR-derived structure of SC16^20^ (PDB ID: 2NBH; grey). (**c**) Ribbon representation of 4 symmetrically related SC16 molecules (coloured differently) in the C222_1_ SC16 crystal. The inset highlights a putative biological contact spanning 606.80 Å^2^ that involves portions of L_1_, L_2_, and β-barrel structure from one molecule (coloured cyan and orange) and the N- and C-termini of a second molecule (coloured light blue and red). Residues flagged by EPPIC as participating in close contacts are highlighted orange or red, labelled, and drawn as sticks. The second inset illustrates the association between the N-terminal tail of one SC16 molecule with the β_1_ strand of another. Putative hydrogen bonds are indicated with yellow lines.

Protein crystallography inherently relies on protein association, and proteins will often crystallize in biologically meaningful ways because of favourable energetics^22^. Therefore, we set out to crystallize SC16 in order to gain insight into which regions of the molecule may form extensive intermolecular contacts and hence may be involved in the self-association that occurs during rodlet self-assembly. After optimizing initial conditions, SC16 crystallized as a cluster of plates over 1–2 weeks (Supplemental Fig. 1). Virtually indistinguishable atomic-resolution structures were determined for two crystals of SC16 that existed in two different space groups: C222_1_ (Fig. 1 light blue; 2.0 Å resolution) and P2_1_2_1_2 (Fig. 1b dark blue; 2.2 Å resolution). These structures superpose to a root mean squared deviation (RMSD) of 0.1 Å and had refinement statistics consistent with good quality models (Table 1). The SC16 crystal structure consists of four disulphide bonds which are characteristic of hydrophobins^18^, a β-barrel core consisting of four β-strands (β_1_: 39Tyr-46﻿Asp, β_2_: 69Leu-76Pro, β_3_: 92Val-98Tyr, β_4_: 102Leu-107Cys), and three loops (L_1_-L_3_) that connect the β-strands. An α-helix was present in L_1_ from 50Lys-59Leu. Residues near the N-terminus and L_2_ (C222_1_: 79Val-85Asn, P2_1_2_1_2: 78Ser-85Asn) did not have interpretable electron density, consistent with these regions being flexible. Overall, the structure of SC16 obtained from crystallization is consistent with the NMR-derived ensemble of SC16 structures^20^ (Protein Data Bank, PDB ID:2NBH, RMSD of 1.39 Å and 1.37 Å for the C222_1_ and P2_1_2_1_2 crystal forms, respectively, Fig. 1b).

**Table 1:** X-ray diffraction data collection and refinement statistics.

PDB ID	7S86	7S7S
**Data collection**		
Space group	C 2 2 2_1_	P 2_1_ 2_1_ 2
Wavelength (Å)	1.54	1.54
Unit cell dimensions		
a, b, c, (Å)	49.2, 96.3, 37.9	48.5, 37.7, 43.8
α, β, γ (°)	90, 90, 90	90, 90, 90
Resolution (Å)	48.2–2.0 (2.07–2.0)	21.9–2.2 (2.28–2.20)
R_measure_ (%)	0.05 (0.35)	0.20 (0.80)
*I/σI*	43.5 (7.9)	21.4 (6.3)
*CC*_*1/2*_	0.99 (0.99)	0.99 (0.98)
Completeness (%)	99.3 (97.5)	100.0 (100.0)
Redundancy	11.5 (11.1)	58.3 (58.3)
No. of reflections	72,580 (6598)	255,148 (25,083)
Unique reflections	6318 (596)	4374 (422)
**Refinement**		
Resolution (Å)	48.1–2.2 (2.52–2.20)	21.9–2.20 (2.52–2.20)
R_work_/R_free_	0.202/0.249 (0.227/0.274)	0.171/0.229 (0.153/0.244)
No. of atoms		
Protein	1197	1168
Water	50	63
*B*-factors (Å^2^)		
Protein	35.7	25.5
Water	45.2	32.6
R.m.s deviations		
Bond lengths (Å)	0.004	0.011
Bond angles (°)	0.628	1.166
Ramachandran statistics		
Favored (%)	93.67	92.21
Allowed (%)	6.33	7.79
Outliers (%)	0.00	0.00

We then used the EPPIC (Evolutionary Protein–Protein Interface Classification) webserver^23^ to probe for large protein–protein interfaces in the crystal lattice that may represent biologically relevant associations of SC16. EPPIC detected a large intermolecular interface that was present in both SC16 crystals and had a surface area of 607 Å^2^ and 589 Å^2^ for the C222_1_ and P2_1_2_1_2 space groups, respectively (Fig. 1c). This surface involves both the N- and C-termini of SC16 interacting with L_1_, L_2_, and the β-barrel of a second symmetry-related SC16 molecule, with 44Thr, 72Leu, 89Ala, and 110Ile being flagged by EPPIC as participating in close contacts.

In particular, the N-terminal tail adopts an extended structure from 27Gly-32Ser with partial β-strand character that associates with the β_1_ strand over residues 42Asn-46Asp of a symmetry related SC16 molecule (Fig. 1c). Although the buried surface area (~ 600 Å^2^) is smaller than the 800 Å^2^ expected for a definitive biological contact^24^, it is larger than a typical crystal contact, involves a very small protein, and is found in two different space groups. Repeating units of this interface also result in a linear association of SC16 monomers that suggests a possible assembly pattern within rodlets.

### NMR spectroscopy identifies the termini and L_2_ of SC16 as dynamic

**Figure 2:**
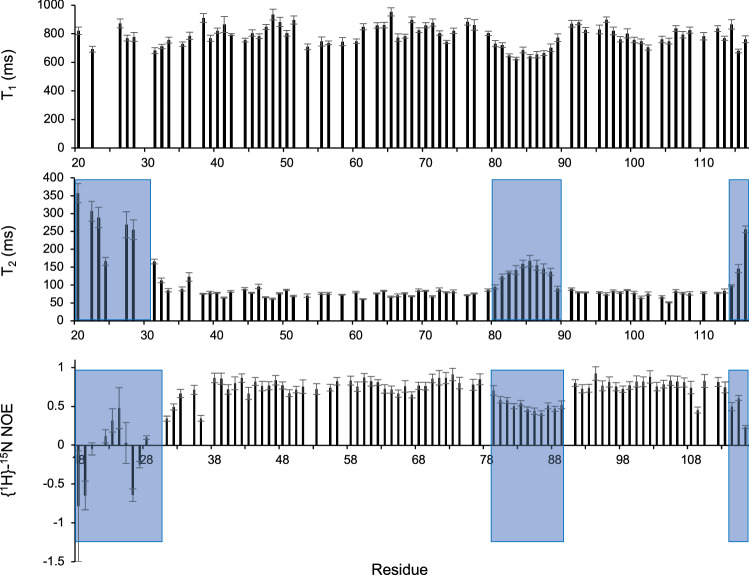
T1, T2, and NOE relaxation measurements identify the N- and C-termini and L_2_ of SC16 as dynamic. T_1_, T_2_, and NOE measurements were collected for ^15^N^13^C-labelled SC16 and are plotted on a per-residue basis. Residues that have elevated T_2_ and decreased NOE values relative to the rest of the molecule are indicated with blue shading.

In both crystal structures of SC16, sections of the N-terminus and L_2_ were poorly defined. To assess the flexibility of SC16 in solution we used NMR spectroscopy to determine which regions of the protein are dynamic. Through analysis of triple resonance NMR spectra and with the aid of deposited NMR assignments for SC16 (BioMagRes Bank accession 25976)^20^ we were able to assign 89% of the amide resonances of SC16. To assess the dynamics of SC16 on the ns-ps timescale under native conditions, T_1_ (longitudinal) relaxation, T_2_ (transverse) relaxation, and heteronuclear nuclear Overhauser effect (NOE) experiments were collected and analyzed using the relaxation module of CcpNmr Analysis (Fig. 2). Values of T_1_ were constant over the entire protein with an average value of 808 ± 66 ms, while T_2_ and NOE values both plateaued over the ranges of 31Lys–﻿79Val and 89Ala–﻿112Ile, with values of 82 ± 18 ms and 0.748 ± 0.014, respectively. T_2_ values increased and NOE values decreased for the N- and C-termini and L_2_ (80Ile–88Ser), identifying these as dynamic regions that may not be resolvable by X-ray diffraction. Importantly, this suggests that 27Gly-32Ser are dynamic enough to undergo conformational changes and be important for rodlet assembly of SC16. Based on the dynamic nature of the N-terminus and its involvement in a large, possibly biological, crystal contact, we generated a variant of SC16 that lacked the N-terminus up to 31Lys (SC16ΔN). SC16ΔN was expressed and purified in the same manner as SC16 (Supplemental Figs. 2 and 3).

### The N-terminal tail of SC16 is dispensable for rodlet assembly

**Figure 3:**
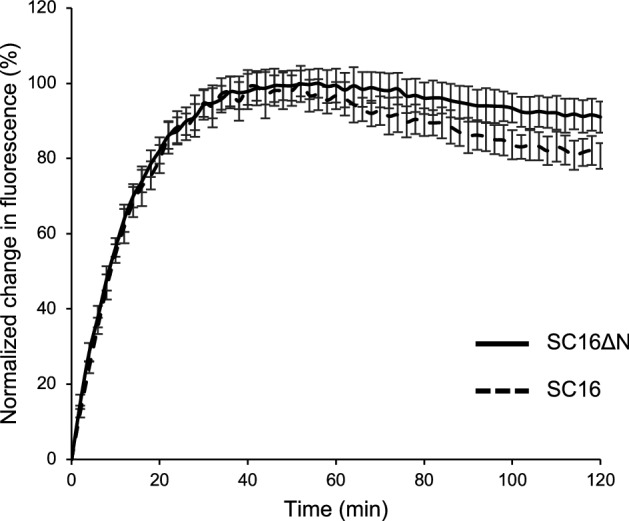
SC16ΔN self-assembles into β-sheet containing rodlets. Thioflavin T fluorescence assays were used to monitor the assembly of SC16 (dashed line) and SC16ΔN (solid line) during agitation. ThT assays were carried out at pH 8.5 with 1 M NaCl. Error bars represent standard deviation from 6 replicates.

Thioflavin T (ThT) is a fluorescent dye commonly used to quantify amyloid formation and hydrophobin assembly, since fluorescence emission intensity is linearly related to fibril concentration^25^. Thus, ThT fluorescence assays were used to quantitatively measure the assembly of both SC16 and SC16ΔN when agitated to create an air:water interface. SC16ΔN assembly was monitored in buffers of pH 5.5–8.5 containing 10 mM–1 M NaCl (Supplemental Fig. 4). Among these conditions, pH 8.5 and 1 M NaCl generated maximal ThT fluorescence upon SC16ΔN agitation. In this buffer condition, the assembly kinetics of SC16 and SC16ΔN were indistinguishable (Fig. 3), suggesting that residues 18Thr-30Pro do not play a significant role in the rate of SC16 self-assembly.

**Figure 4:**
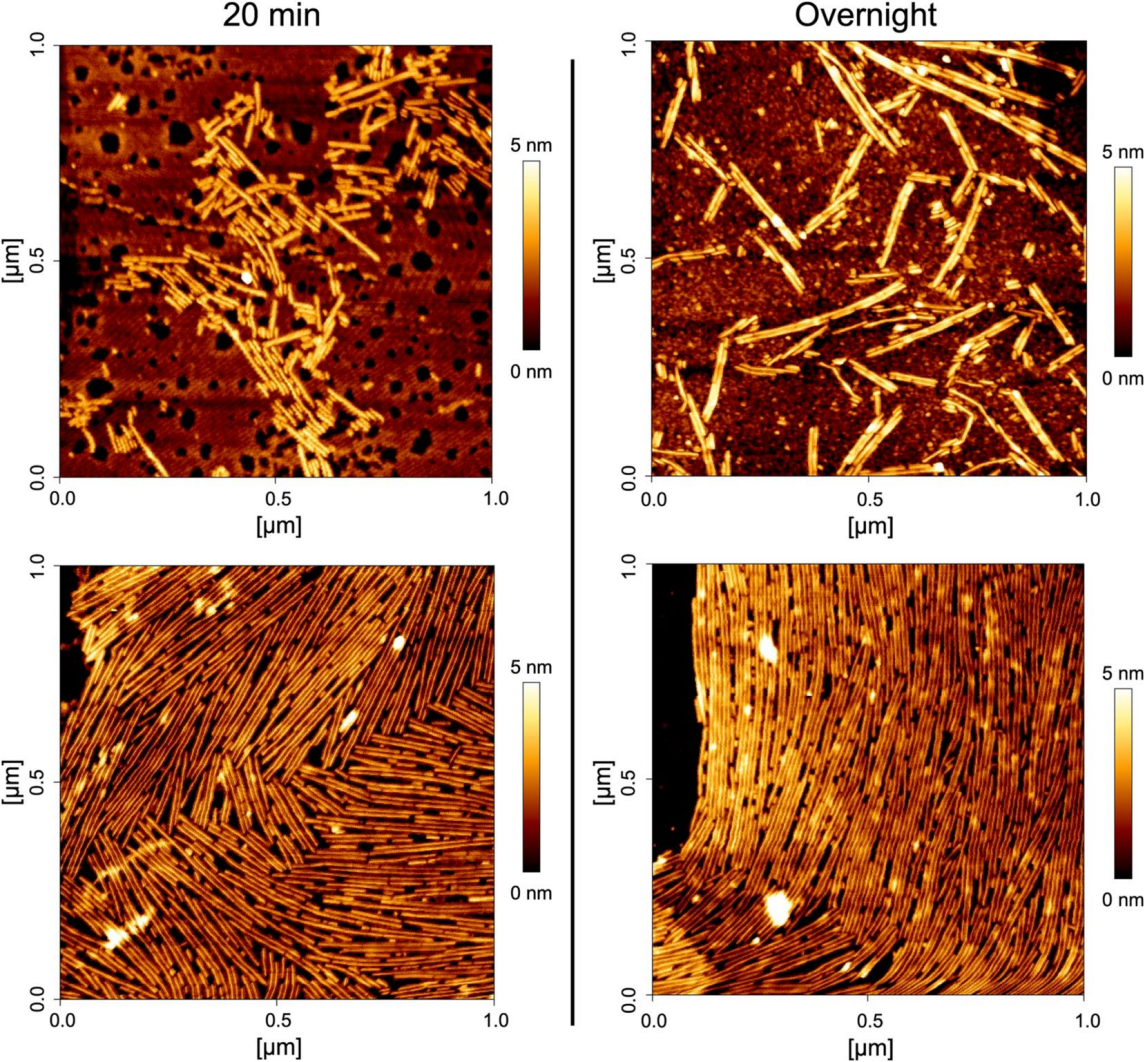
SC16ΔN self-assembles into rodlets. A 50 μL drop of SC16 ΔN in dH_2_O (left: 10 μg/mL; right: 5 μg/mL) was incubated on freshly cleaved highly oriented pyrolytic graphite at room temperature for (left) 20 min before wicking excess solution and drying overnight or (right) allowing to dry overnight. Rodlets were then imaged using atomic force microscopy and a representative image from two samples for each condition is shown.

We further characterized the morphology of SC16ΔN rodlets with atomic force microscopy. SC16ΔN was deposited onto freshly cleaved highly oriented pyrolytic graphite (HOPG) and allowed to assemble for either 20 min or overnight (Fig. 4 with additional fields shown in Supplemental Fig. 5). Rodlets of a uniform width (~ 10 Å) were observed and were often paired together. The rodlets could either remain dispersed (Fig. 4, top) or form laterally associated bundles (Fig. 4, bottom), which is consistent with what has been observed previously for SC16^20^ and rodlets from other hydrophobins^21,26^. Interestingly, the longest rodlets often form from multiple shorter rodlets closely organized end-to-end.

Understanding the mechanism of and the sequences responsible for hydrophobin assembly is important for developing commercial applications of these proteins. For example, mutation of residues important for assembly could tailor hydrophobin assembly to different buffer conditions. Conversely, regions that are not important for self-assembly can be modified to add new functionality to hydrophobins. The mechanistic origins of hydrophobin rodlet assembly are unclear and they seem to vary depending on the hydrophobin studied. For example the class IA hydrophobin EAS requires sequences in L_3_ for assembly^21^, while sequences in L_1_ are dispensable^27^. Conversely in the hydrophobin DewA (from *Aspergillus nidulans*), L_2_ is predicted to be required for self-assembly and rodlet formation^26^.

In this study we used X-ray crystallography to determine the structure of SC16 and investigated a potential mode of its self-assembly. We found that although the N-terminal tail of SC16 associates with neighbouring molecules in the crystalline state, it is dynamic in solution and does not significantly influence the self-assembly rate or morphology of SC16 rodlets. This suggests that other regions of SC16 (such as L_1_ and L_2_) are likely essential for rodlet formation. These results outline an opportunity for hydrophobin functionalization, since modification of the N-terminal tail of SC16 should be a viable strategy to create functionalized SC16 variants that are still able to self-assemble onto surfaces.

## Methods

### Plasmid construction

pET21-derived plasmids coding for SC16 with the N-terminal signal sequence removed (18Thr–114Leu of the *hyd1* gene of *S. commune*; NCBI ID: EFI94929) with upstream sequences coding for a hexahistidine tag, the B1 domain of protein G, and a thrombin protease recognition sequence (H_6_-GB1-SC16) are the same as those used previously^28^. A second expression plasmid was constructed using restriction enzyme-based procedures that coded for SC16 with 13 residues from the N-terminal tail removed (residues 31Lys-114Leu of SC16; H_6_-GB1-SC16ΔN). The validity of all plasmids was verified by sequencing (Eurofins Genomics).

### Protein expression and purification

SC16 and SC16ΔN were isolated as previously described for SC16^28^. Briefly, Shuffle T7 express *E. coli* (New England Biolabs) were transformed with expression plasmids. After overnight expression at 20 °C, cells were lysed via resuspending in lysis buffer (20 mM Tris pH 8, 250 mM NaCl) and heating to 80 °C for 15 min. Hydrophobins were isolated via Ni^2+^ affinity chromatography and then cleaved overnight with thrombin while being dialyzed against 20 mM Tris pH 8, 50 mM NaCl, 1 mM CaCl_2_. After cleavage SC16 and SC16ΔN were isolated by Ni^2+^ affinity chromatography, ion exchange chromatography, and finally by size exclusion chromatography in 20 mM Tris pH 8, 50 mM NaCl. For NMR studies of SC16, ^15^N- or ^13^C/^15^N-labelled SC16 was produced by growing cells in ^15^N or ^15^N/^13^C-enriched M9 minimal media (adapted from Studier)^29^, and purified as above. After purification samples were either used immediately or frozen at − 20 °C for long-term storage.

### Crystallography

SC16 was used for crystallization trials immediately after purification. Initial crystallization screens were performed using a sitting drop setup and the MCSG1-4 crystallization screens (Anatrace). After optimization, crystals were grown by the hanging drop vapour diffusion method at 22 °C. Drops were 4 μL and contained 1:1 protein (14.15 mg/mL) to reservoir (0.1 M citric acid pH 3.5 with either 30.5% or 29% w/v PEG3350) solution. Crystals grew as plates over 1–2 weeks after which individual crystals were cryoprotected with reservoir solution containing 25% ethylene glycol. Data for the crystal grown in 30.5% PEG3350 was collected at 100 K at the Canadian Light Source (08B1-1 beamline)^30,31^. Data for the crystal grown in 29% PEG 3350 was collected at 100 K using a D8 Quest diffractometer with Cu microfocus source (Bruker AXS). Molecular replacement was used to phase both datasets using the Phenix software package and the NMR-derived structure of SC16 as the search model (PBD ID: 2NBH)^20,32^. Further model refinement was completed using Phenix^32^ and Coot^33^ and final structures are deposited in the Protein Data Bank^34^ (PDB IDs 7S86 and 7S7S for SC16 in the C222_1_ and P2_1_2_1_2 space groups, respectively). The Pymol Molecular Graphics System (v2.1, Schrödinger) was used for model visualization and preparing figures. The EPPIC (Evolutionary Protein–Protein Interface Classification) webserver was used to probe for large protein–protein interfaces in the crystal lattice^23^.

### Nuclear magnetic resonance spectroscopy

A 300 μM sample of ^13^C/^15^N-labelled SC16 in 20 mM MES pH 6.5, 50 mM NaCl, 5% D_2_O was prepared. ^1^H–^15^N-heteronuclear single quantum coherence (HSQC), HNCA, HNCACB, HN(CO)CA, and CBCA(CO)NH experiments were collected using a 700 MHz Avance III spectrometer (National Research Council Institute for Marine Biosciences, Halifax, NS) and used to assign backbone resonances of SC16^35^. T_1_ and T_2_ relaxation, as well as heteronuclear nuclear Overhauser effect (NOE) experiments were collected using an 800 MHz Bruker Avance III HD console and Ascend magnet with TCI cryoprobe (the Québec/Eastern Canada High Field NMR Facility (QANUC)) and analyzed using the relaxation module of CcpNmr Analysis^36^.

### Thioflavin T assay

SC16ΔN and SC16 were dialysed against dH_2_O over night at 4 °C before the assay. Reactions for SC16ΔN were set up in a matrix of buffer conditions consisting of a multicomponent buffer (20 mM each of MES, Tris, phosphate) set to varying pH (5.5, 6.5, 7.5, 8.5). Assembly at each pH was measured in the presence of 10 mM, 250 mM, and 1 M NaCl. SC16 reactions were set up only for pH 8.5, 1 M NaCl. The total reaction volumes were 100 μL and all conditions contained 50 μg/mL protein, 20 μM filtered ThT. Each condition was replicated 6 times and measured in a sealed 96-well black-bottomed plate. A SpecraMax M3 plate reader was used to monitor the ThT fluorescence (λ_ex_ = 430 nm, λ_cutoff_ = 455 nm, and λ_em_ = 480 nm) while shaking the reactions for 30 s every 2 min.

### Atomic force microscopy

SC16ΔN was dialysed against dH_2_O overnight at 4 °C. Hydrophobin drops (50 µL at 5 µg/mL) were placed onto freshly cleaved 3 mm HOPG discs and allowed to dry overnight. Alternatively, 50 µL drops containing 10 μg/mL of hydrophobins were allowed to sit on the HOPG for 20 min. Excess solution was wicked away and HOPG was allowed to dry overnight at room temperature. Two samples were produced for each condition and at least 3 fields of view were collected for each sample. Images were collected on a NanoWizard II Ultra from JPK using Tap300AI-G tips (Budget Sensors).

## Supplementary Information

Supplementary Information.

## Abbreviations

NMR: Nuclear magnetic resonance
RMSD: Root mean squared deviation
PDB: Protein data bank
NOE: Nuclear Overhauser effect
ThT: Thioflavin T
HOPG: Highly oriented pyrolytic graphene
