# Supplementary material for: The N-terminal tail of the hydrophobin SC16 is not required for rodlet formation
Source: Sci Rep. 2022 Jan 10;12:366. doi: 10.1038/s41598-021-04223-6 (PMC8748815; doi:10.1038/s41598-021-04223-6)
Supplement: Supplementary file 1 — Supplementary Information. [file 41598_2021_4223_MOESM1_ESM.pdf]

Supplemental information for:

The N-terminal tail of the hydrophobin SC16 is not required for rodlet formation

Kathleen L. Vergunst and David N. Langelaan

Contents:

Figure S1 **SC16 crystals grew as clusters of plates** 2
Figure S2 **Expression and purification of SC16** 3
Figure S3 **Expression and purification of SC16ΔN** 4
Figure S4 **Thioflavin T analysis of SC16ΔN self-assembly** 5
Figure S5 **Atomic force microscopy of SC16ΔN rodlets** 6

a

b

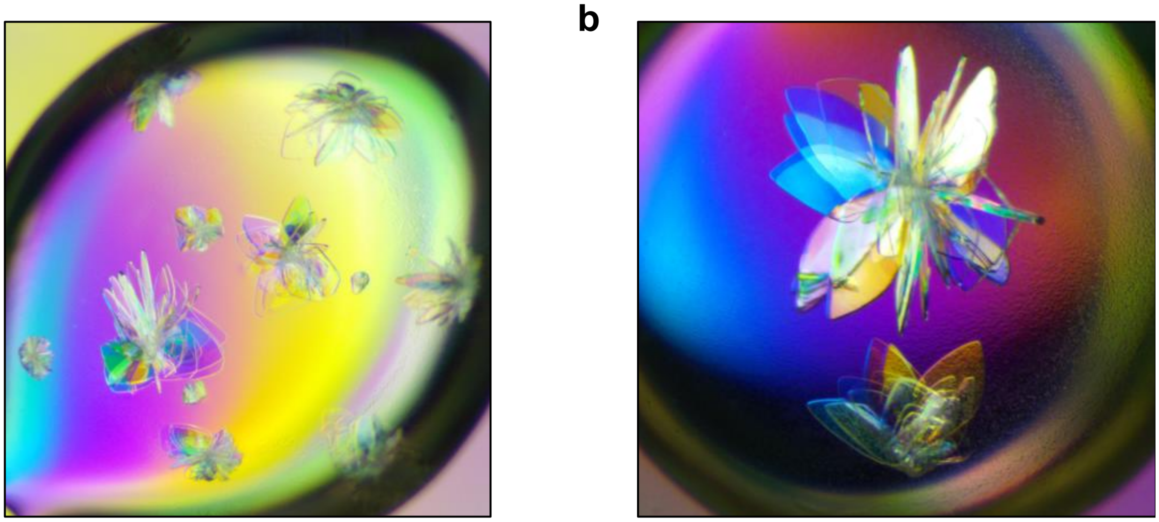

Figure S1. SC16 crystals grew as clusters of plates. Images of representative crystals of SC16 in **(a)** $C222_1$ and **(b)** $P2_12_12$ space groups. Crystals were imaged under polarized white light.

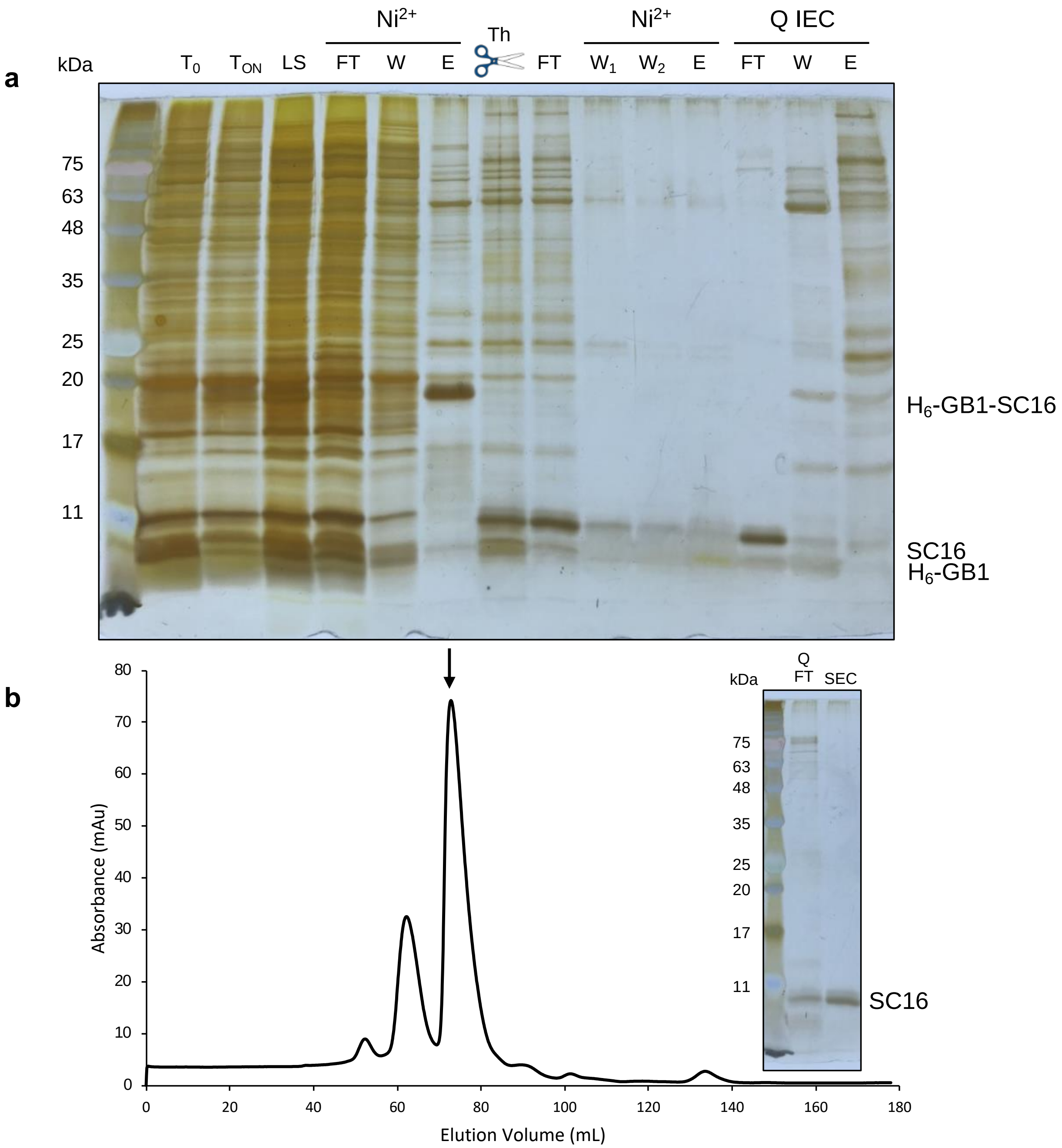

Figure S2. Expression and purification of SC16. **a)** SDS-PAGE analysis of SC16 purification. SC16 was expressed in SHuffle *E. coli* and following induction with isopropyl 1-thio-β-D-galactopyranoside (T_0), grown overnight at 20°C (T_{ON}), lysed by hot water bath, and the supernatant was collected (LS) prior to Ni^{2+} affinity chromatography. After protease cleavage (Th), SC16 was next purified by a second round of Ni^{2+} affinity chromatography, with the resulting flow through and wash further purified by ion exchange chromatography (Q). Fractions shown: FT: Flow Through, W: Wash, and E: Elution. SC16 is indicated and has an expected molecular mass of 10.23 kDa. **b)** Size exclusion chromatogram of purified SC16. SC16 is indicated by an arrow and purity was assessed by SDS-PAGE (inset). Silver staining was used to visualize SC16.

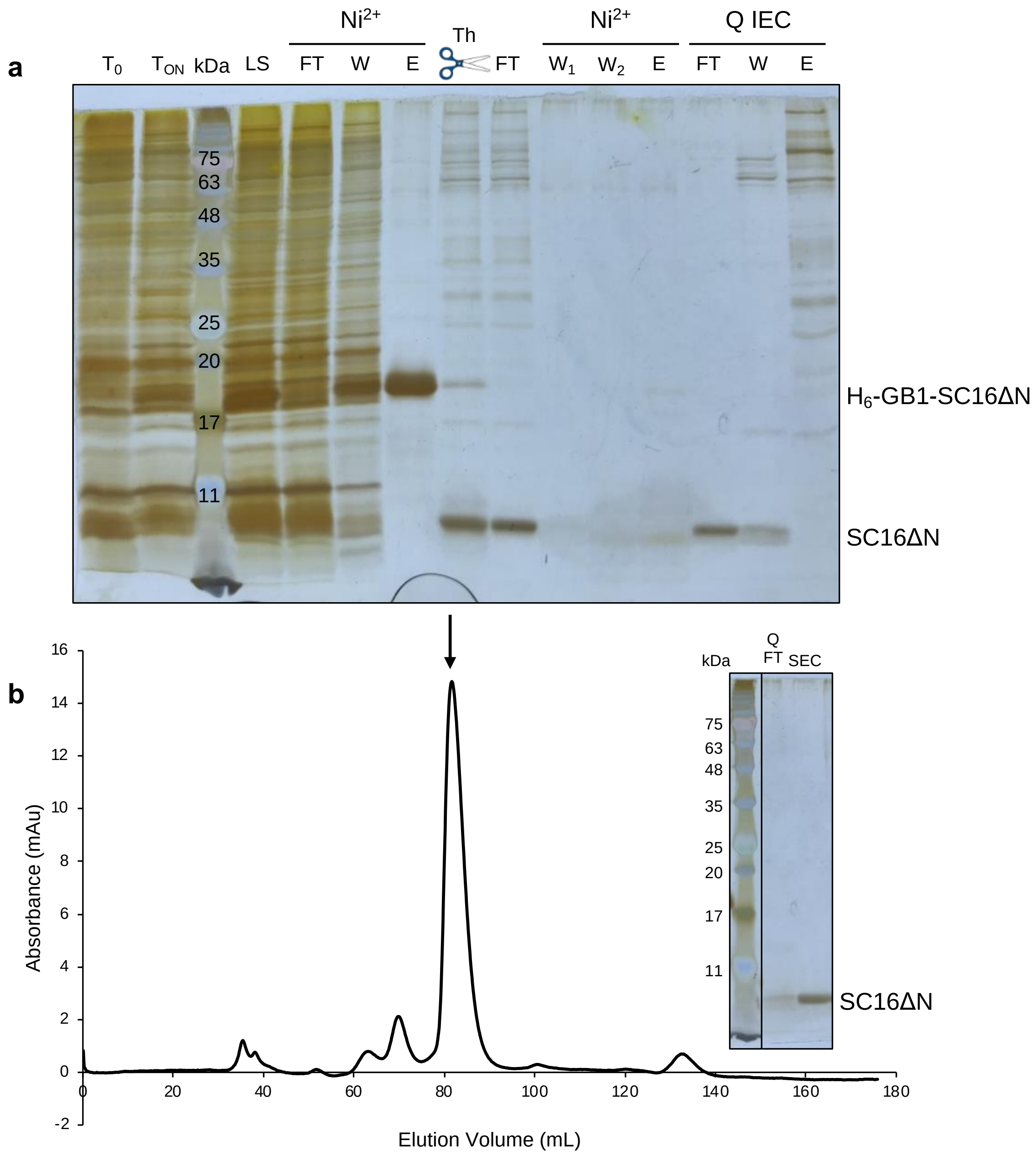

Figure S3. Expression and purification of SC16ΔN. **a)** SDS-PAGE analysis of SC16ΔN purification. SC16ΔN was expressed in SHuffle *E. coli* and following induction with isopropyl 1-thio-β-D-galactopyranoside (T_0), grown overnight at 20°C (T_{ON}), lysed by hot water bath, and the supernatant was collected (LS) prior to Ni^{2+} affinity chromatography. After protease cleavage (Th), SC16ΔN was next purified by a second round of Ni^{2+} affinity chromatography, with the resulting flow through and wash further purified by ion exchange chromatography (Q). Fractions shown: FT: Flow Through, W: Wash, and E: Elution. SC16ΔN is indicated and has an expected molecular mass of 8.97 kDa. **b)** Size exclusion chromatogram of purified SC16ΔN. SC16ΔN is indicated by an arrow and purity was assessed by SDS-PAGE (inset). Silver staining was used to visualize SC16ΔN.

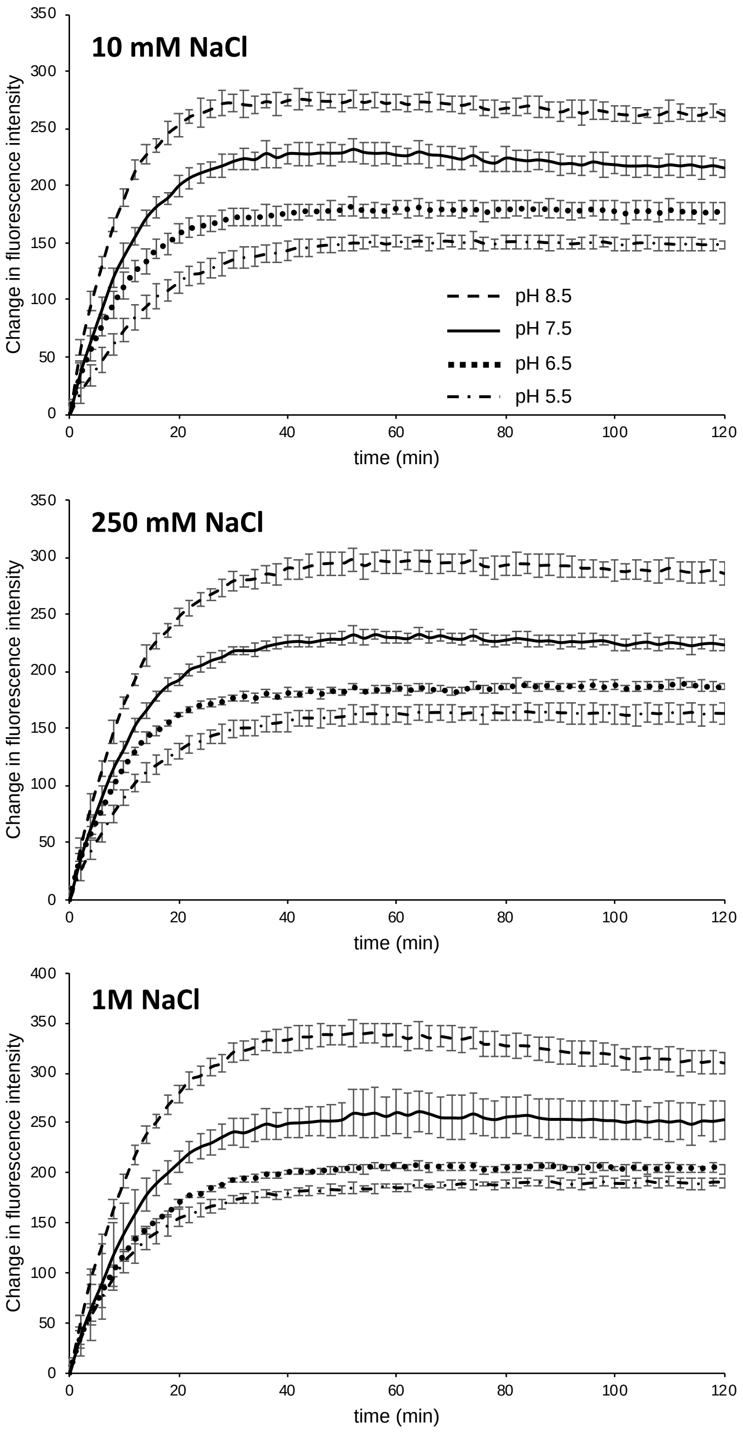

Figure S4. Thioflavin T analysis of SC16ΔN self-assembly. Self-assembly of SC16ΔN in a buffer matrix (pH 5.5, 6.5, 7.5, 8.5; [NaCl] 10 mM, 250 mM, 1 M) was monitored by ThT fluorescence. Samples were monitored over 2 hours every 2 minutes with 30 seconds of mixing between measurements. Error bars represent standard deviation from 6 replicates.

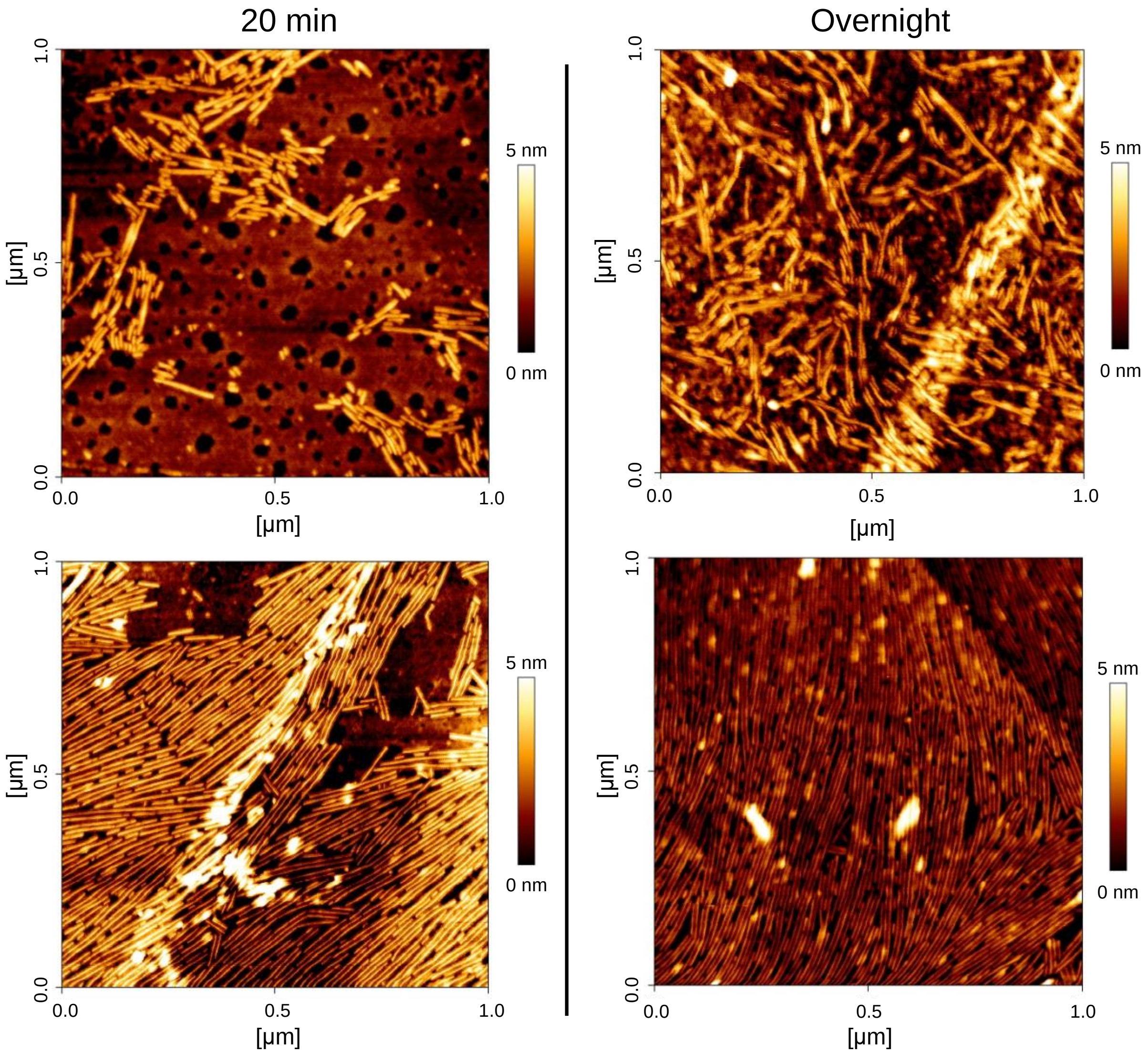

Figure S5. Atomic force microscopy of SC16ΔN rodlets. Different regions of the surfaces coated with assembled SC16ΔN that were imaged in Fig. 4. A 50 μL drop of SC16ΔN in dH_2O (**left**: 10 μg/mL; **right**: 5 μg/mL) was incubated on freshly cleaved highly oriented pyrolytic graphite at room temperature for **(left)** 20 min before wicking excess solution and drying overnight or **(right)** allowing to dry overnight. These images were collected using a NanoWizard II Ultra with Tap300AI-G tips.
